# Non-invasive prenatal diagnosis by massively parallel sequencing of maternal plasma DNA

**DOI:** 10.1098/rsob.120086

**Published:** 2012-06

**Authors:** Yuk Ming Dennis Lo

**Affiliations:** 1 Li Ka Shing Institute of Health Sciences, The Chinese University of Hong Kong, Prince of Wales Hospital, Shatin, New Territories, Hong Kong SAR, People's Republic of China; 2 Department of Chemical Pathology, The Chinese University of Hong Kong, Prince of Wales Hospital, Shatin, New Territories, Hong Kong SAR, People's Republic of China

**Keywords:** Down syndrome, trisomy 21, plasma nucleic acids, foetal DNA in maternal plasma, next-generation sequencing

## Abstract

The presence of foetal DNA in the plasma of pregnant women has opened up new possibilities for non-invasive prenatal diagnosis. The use of circulating foetal DNA for the non-invasive prenatal detection of foetal chromosomal aneuploidies is challenging as foetal DNA represents a minor fraction of maternal plasma DNA. In 2007, it was shown that single molecule counting methods would allow the detection of the presence of a trisomic foetus, as long as enough molecules were counted. With the advent of massively parallel sequencing, millions or billions of DNA molecules can be readily counted. Using massively parallel sequencing, foetal trisomies 21, 13 and 18 have been detected from maternal plasma. Recently, large-scale clinical studies have validated the robustness of this approach for the prenatal detection of foetal chromosomal aneuploidies. A proof-of-concept study has also shown that a genome-wide genetic and mutational map of a foetus can be constructed from the maternal plasma DNA sequencing data. These developments suggest that the analysis of foetal DNA in maternal plasma would play an increasingly important role in future obstetrics practice. It is thus a priority that the ethical, social and legal issues regarding this technology be systematically studied.

## 2. Introduction

Prenatal diagnosis is now an established part of modern obstetrics practice. However, conventional definitive methods for prenatal diagnosis involve the invasive sampling of foetal tissues, using methods such as amniocentesis and chorionic villus sampling (CVS). Such methods carry with them a small, but definite risk for the foetus [1]. Ultrasound scanning and maternal serum biochemical analysis have emerged as non-invasive methods for screening for foetal chromosomal aneuploidies, such as trisomy 21 [2]. However, for the detection of foetal chromosomal aneuploidies, such methods measure epiphenomena, which are associated with the disorders, rather than analysing the core pathology. As a result, they typically can only be used within a relatively narrow gestational age window, and despite their development over many years, their sensitivity and specificity still have much room for improvement.

Because of these limitations, there has been a search over the last few decades for safe, non-invasive methods for prenatal diagnosis that can allow the direct analysis of foetal genetic materials. Early work had focused on the isolation of foetal nucleated cells that had entered into the maternal blood. However, the concentrations of such cells are very low, typically of the order of one or a few foetal nucleated cells per millilitre of maternal blood [3]. Probably as a result of such low concentrations, prenatal testing carried out using circulating foetal cells has been found to have relatively low sensitivity and specificity [4].

In 1997, Lo *et al.* were inspired by the presence of tumour-derived DNA in the plasma and serum of cancer patients [5,6], and wondered whether an analogous phenomenon might also be present in pregnancy. Lo *et al*. were able to find Y chromosomal DNA sequences in the plasma and serum of women carrying male foetuses, and concluded that cell-free foetal DNA was present in maternal plasma and serum [7]. Subsequent measurements using real-time polymerase chain reaction (PCR) have indicated that cell-free foetal DNA is present in maternal plasma at a mean fractional concentration of 3 per cent in the first and second trimesters [8]. More recent measurements using more accurate digital PCR-based techniques show that the median fractional concentration may be approximately 10 per cent [9]. Such relatively high fractional foetal DNA concentrations, when compared with the fractional concentrations of foetal nucleated cells in maternal blood, suggest that non-invasive prenatal diagnosis carried out using the former would probably be more robust. Another advantage of the use of foetal DNA in maternal plasma, compared with circulating foetal nucleated cells, is the lack of persistence of the former following delivery [10,11]. Conversely, there are numerous reports describing the persistence of foetal nucleated cells in the maternal circulation following delivery [12].

The robustness of non-invasive prenatal diagnosis using cell-free foetal DNA in maternal plasma can be seen by the many reports that describe diagnostic tests based on the detection of DNA sequences that the foetus has inherited from the father and which are absent in the maternal genome. Examples include the detection of foetal sex based on the detection of Y chromosomal DNA from maternal plasma [13] and the detection of the *RHD* gene of a Rhesus D-positive foetus in the plasma of a Rhesus D-negative pregnant woman [14,15].

## 3. The challenge for the detection of foetal chromosomal aneuploidies

The detection of foetal chromosomal aneuploidies, such as trisomy 21, is much more challenging than the determination of foetal sex and Rhesus D blood group genotype because, apart from detecting the presence of foetal DNA in maternal plasma, one also has to measure the foetal chromosome dosage involving the chromosome of interest. The latter task is made more difficult because of the fact that foetal DNA is present as a minor fraction of the DNA that is found in maternal plasma [8,9].

Early work has focused on the analysis of a subset of nucleic acids present in maternal plasma that is foetal-specific. Examples include DNA molecules bearing foetal-specific DNA methylation patterns [16–19] and RNA molecules that are specifically transcribed from the placenta [20]. All but one [19] of the above-mentioned methods involve the use of genetic polymorphisms and necessitate the use of multiple markers to achieve a broad population coverage. The method that does not require the use of genetic polymorphisms is based on chromatin immunoprecipitation and complex data normalization procedures [19]. These steps would be challenging to be reproducibly performed for a clinical diagnostic or a screening test. Another approach that has been described involves the enrichment of the fractional concentration of foetal DNA. One way to achieve such enrichment uses the observation that foetal DNA molecules in maternal plasma are shorter than the maternally derived ones [21,22]. However, the amount of enrichment that one could achieve thus far using this approach is relatively limited. Another method that has been reported involves the use of formaldehyde on maternal blood samples. This method has been claimed to minimize the release of DNA from the maternal blood cells and thus to result in a higher fractional concentration of foetal DNA in the plasma fraction [23,24]. However, this method has not been reproduced by a number of laboratories [25,26].

## 4. Molecular counting approach

An alternative approach for the detection of foetal chromosomal aneuploidies is to measure the quantitative perturbations in the genomic representation of the involved chromosome in maternal plasma. However, the challenge is that such perturbations are generally very small and are related to the fractional concentration of circulating foetal DNA [27]. For example, it has been shown that when the fractional foetal DNA concentration is 10 per cent, the genomic representation of chromosome 21 in maternal plasma will be increased by 5 per cent by the presence of a trisomy 21 foetus. The detection of such a small amount of quantitative perturbation requires the use of extremely precise measurement methods. In 2007, it was demonstrated that single-molecule counting techniques using digital PCR as an example can be used for such a purpose [27,28]. Such an approach was first realized using plasma foetal (placenta-derived) RNA that is present in maternal plasma [27]. The potential extension of this approach to plasma DNA was also explored using DNA mixtures and computer simulations [27,28]. The numbers of molecules that one would need to be counted for different fractional foetal DNA concentrations have been outlined. For example, it is suggested that to achieve the detection of a trisomy 21 foetus in a maternal plasma sample containing 25 per cent foetal DNA would require the performance of 7680 digital PCRs. Furthermore, for every twofold reduction in the fractional concentration of foetal DNA, the number of molecules that one would need to count would increase by 2^2^ (i.e. 4) times [27].

## 5. Non-invasive prenatal diagnosis by massively parallel sequencing

With the advent of massively parallel sequencing, it has become relatively easy to count millions (or even billions) of DNA molecules [29]. In 2008, two groups showed that the massively parallel sequencing of maternal plasma DNA would allow one to work out the genomic representations of different chromosomes in maternal plasma and to detect the perturbations of such representations when a pregnant woman is carrying a trisomic foetus [30,31]. This approach involves the random sequencing of millions of DNA molecules in maternal plasma. Individual sequence tags are aligned to the human genome to determine the chromosome of origin of a particular sequence tag. The sensitivity and specificity of these early reports, involving a relatively small number of samples, are both 100 per cent. It has been shown that both sequencing-by-synthesis [30,31] and sequencing-by-ligation [32] platforms would work for this approach. The method is also applicable for foetal trisomy 21 that is caused by a Robertsonian translocation [33].

The throughput of the use of massively parallel sequencing for the detection of foetal trisomy 21 has since then been validated by a number of large-scale clinical studies [34–37]. The detection of foetal trisomies 13 and 18 has been shown to be more challenging owing to the GC contents of the involved chromosomes and the analytical bias of the sequencing platform in relation to the GC contents [31,32]. Nonetheless, the use of bioinformatics algorithms that correct for such bias has been shown to allow the improved detection of trisomies 13 and 18 [38,39]. The robust detection of trisomies 13 and 18 has recently been validated by a number of large-scale studies [37,40,41]. The future use of single-molecule sequencing platforms that do not require the use of an amplification step might further reduce the influence of the GC content and might improve the robustness of this approach [42].

As a result of the various large-scale studies, the use of massively parallel sequencing of maternal plasma DNA for the prenatal detection of foetal chromosomal aneuploidies has been introduced as a clinical service in the USA and parts of Asia. It is likely that this technology will be used clinically in other regions around the world in the near future.

The cost of this technology is still relatively expensive when compared with conventional prenatal screening procedures, but is comparable with the costs of invasive testing involving amniocentesis. A number of groups have explored the possibility of reducing the sequencing costs. Random sequencing is the protocol employed by most workers in the field, whereby sequence tags will be obtained for all chromosomes [30,31]. The number of sequence tags obtained per chromosome is proportional to the size of each chromosome. In such a protocol, only a proportion of the sequenced reads will align to the chromosome of interest (e.g. chromosome 21).

In an effort to reduce the cost of sequencing, Liao *et al.* [43] have demonstrated that a solution-based target capture system is able to focus the sequencing power to selected genomic regions. Such a system is able to increase the sequencing coverage of the targeted region by over 200-fold. Another system has recently been described by Sparks *et al.* [44] in which hundreds of sets of oligonucleotides have been used to target selected regions of the genome. These targeted regions are then amplified and sequenced by massively parallel sequencing. Following a process of normalization, the authors have reported that trisomy 21 and 18 samples are distinguishable from euploid samples. However, it is important to note that the authors have only reported a training dataset, but have not tested the robustness of their algorithm using a validation dataset. In a subsequent publication by the same group, the authors have introduced a new algorithm whereby the fractional concentrations of foetal DNA in maternal plasma are incorporated into the data analysis step [45]. Nonetheless, they have not compared the performance of this new algorithm with that of their previous algorithm [44]. The new algorithm, on the other hand, has been tested in an independent cohort [46]. The relative robustness of the selective chromosome sequencing approach [46] and the previously described random sequencing approach [30,31,34,36,37,39,41] would need to be evaluated in future studies. The cost-effectiveness issue of the targeted versus the non-targeted approach also needs to be addressed in the context of the continual and rapid reduction in the costs associated with sequencing.

## 6. Towards prenatal foetal whole genome sequencing

In addition to the detection of foetal chromosomal aneuploidies, the analytical precision offered by molecular counting approaches such as digital PCR has implications for the non-invasive prenatal diagnosis of monogenic diseases. For example, in the situation of a mother who is a heterozygous carrier of an autosomal recessive monogenic disease, the relative dosage of the mutant and normal versions of the gene in the mother's genome should be 1 : 1. However, when the mother is pregnant with a foetus, then the relative dosage of the two versions of the gene in the mother's plasma will be modified depending on the foetal genotype. If the foetus is homozygous for the mutant gene, then the addition of two doses of the mutant gene per genome-equivalent of foetal DNA into the maternal plasma will bias the relative dosage in favour of the mutant gene. If the foetus is homozygous for the normal gene, then the relative dosage of the mutant and normal genes in the mother's plasma will be biased in favour of the normal gene. Finally, if the foetus is heterozygous, then the 1 : 1 relative dosage of the mutant and normal genes will remain unchanged in the mother's plasma. This concept has been called the relative mutation dosage approach [47], and has been used for the non-invasive prenatal diagnosis of the haemoglobinopathies [47] and haemophilia [48].

With the advent of massively parallel sequencing, this type of concept can be applied on a genome-wide scale. Thus, Lo *et al*. [49] demonstrated that by sequencing maternal plasma DNA to the equivalent of 65-fold haploid genome coverage, a genome-wide genetic and mutational profile of a foetus can be assembled from the sequencing data. The authors have further used the genetic maps of the father and the mother to help assemble the foetal genetic map. The resolution of the foetal genomic map that one could assemble is limited by the resolution of the parental genetic maps. This method thus opens up the possibility that the prenatal diagnosis of multiple genetic diseases could be performed by a single test. The future refinement of targeted sequencing approaches would be expected to reduce the cost of this technology.

## 7. Conclusions

It is probable that non-invasive prenatal diagnosis using foetal DNA in maternal plasma would play an increasingly important role in the future practice of prenatal testing. However, it is important to address the ethical, legal and social issues surrounding such developments [50–52]. The positive side of non-invasive testing is the avoidance of harm to the foetus that would be associated with invasive testing. However, some parties may be concerned by the possibility that the availability of non-invasive testing might ‘encourage’ more pregnant women to undergo testing. Such issues, and others, would need specially designed studies to systematically address them.

## Author profile

**Dennis Lo** is the Director of the Li Ka Shing Institute of Health Sciences, Li Ka Shing Professor of Medicine and Professor of Chemical Pathology at The Chinese University of Hong Kong. He received his undergraduate education from the University of Cambridge, and his Doctor of Medicine and Doctor of Philosophy degrees from the University of Oxford. His research interests focus on the biology and diagnostic applications of cell-free nucleic acids in plasma. In particular, he discovered the presence of cell-free foetal DNA in maternal plasma in 1997 and has since then been pioneering non-invasive prenatal diagnosis using this technology. He has received numerous awards for his research, including a State Natural Sciences Award from the State Council of China (2005), the International Federation of Clinical Chemistry and Laboratory Medicine (IFCC)—Abbott Award for Outstanding Contribution to Molecular Diagnostics (2006), the US National Academy of Clinical Biochemistry (NACB) Distinguished Scientist Award (2006), a Cheung Kong Scholars Achievement Award from the Ministry of Education of China (2006), a Silver Bauhinia Star from the Hong Kong SAR Government and the American Association for Clinical Chemistry (AACC)—NACB Award for Outstanding Contribution to Clinical Chemistry in a Selected Area of Research (2012). He was elected a Fellow of the Royal Society of London in 2011.
